# Molecular Dynamics Study of the Effect of Abrasive Grains Orientation and Spacing during Nanogrinding

**DOI:** 10.3390/mi11080712

**Published:** 2020-07-23

**Authors:** Nikolaos E. Karkalos, Angelos P. Markopoulos

**Affiliations:** Laboratory of Manufacturing Technology, School of Mechanical Engineering, National Technical University of Athens, Heroon Polytechniou 9, 15780 Athens, Greece; nkark@mail.ntua.gr

**Keywords:** nanogrinding, abrasive grains, rake angle, spacing, grinding forces, grinding temperature, chip formation, subsurface damage

## Abstract

Grinding at the nanometric level can be efficiently employed for the creation of surfaces with ultrahigh precision by removing a few atomic layers from the substrate. However, since measurements at this level are rather difficult, numerical investigation can be conducted in order to reveal the mechanisms of material removal during nanogrinding. In the present study, a Molecular Dynamics model with multiple abrasive grains is developed in order to determine the effect of spacing between the adjacent rows of abrasive grains and the effect of the rake angle of the abrasive grains on the grinding forces and temperatures, ground surface, and chip formation and also, subsurface damage of the substrate. Findings indicate that nanogrinding with abrasive grains situated in adjacent rows with spacing of 1 Å leads directly to a flat surface and the amount of material remaining between the rows of grains remains minimal for spacing values up to 5 Å. Moreover, higher negative rake angle of the grains leads to higher grinding forces and friction coefficient values over 1.0 for angles larger than −40°. At the same time, chip formation is suppressed and plastic deformation increases with larger negative rake angles, due to higher compressive action of the abrasive grains.

## 1. Introduction

Ultrahigh precision machining plays an increasingly important role in various contemporary high-end industries due to the necessity of fabricating parts or rendering features on various components with micrometer or nanometer dimensions. The strict requirements for ensuring dimensional accuracy of mechanical parts in an extremely small scale render the procedure of selecting the appropriate manufacturing technique considerably important [1]. Some of the most widely employed techniques for manufacturing in the nanoscale level are related to nanolithography technique, whereas in areas such as the production of high quality and precise lens, ultrahigh precision polishing has been proven adequate for achieving roughness value as low as several nanometers [1,2].

Although the aforementioned techniques, such as nanometric level polishing have been already employed in industrial scale, material removal mechanisms at nanometric level have not been yet studied in every aspect. Moreover, the inability of conducting appropriate experiments due to high cost or lack of adequate equipment leads to the necessity of employing alternative methods for the study of nanoscale machining and abrasive processes, by conducting dedicated and reliable simulations [3]. For the macroscale and microscale counterparts of these processes, the finite element method (FEM) has been successfully employed for decades, as it can achieve a sufficient level of accuracy and has been assistive to the understanding of various underlying phenomena. Despite the fact that by using special models, microscale phenomena can be modeled with FEM, the discrete nature of matter in the nanoscale level and inability of continuum theories to represent atomistic interactions led to the use of Molecular Dynamics (MD) method as a viable alternative for nanoscale simulations.

The MD method, developed originally in the 1950s by Alder and Wainwright [4,5], is an established computational method for studying various phenomena and processes related to the nanoscale, such as the determination of fundamental material structure and properties. This method models directly the atomistic structure of materials and approximates the material behavior in the nanoscale level with a high degree of accuracy. Although in most cases, a direct comparison with experimental data is not feasible, MD method can provide adequate explanations regarding phenomena which have their origins in the nanoscale, such as plastic deformation and material removal mechanism during machining and abrasive process, and thus, can act complementary to macroscale experiments and simulations by supporting their findings. In the field of nanocutting simulations, it has been applied during the last three decades, stemming from the pioneering works of Belak and Stowers [6] and Belak, Boercker, and Stowers [7]. These early studies, although they were conducted with relatively small and simple models, set the path for the more advanced models which were presented during the last three decades. It is worth noting that although most models were simpler than the current ones, the authors distinguished the nanocutting from the nanoabrasive models, which included one or two abrasive grains with a linear motion. During the last decades, considerable progress has been conducted in this field, especially regarding nanogrinding simulations, which will be briefly discussed afterwards.

Inasaki and Rentsch [8,9] were among the first to investigate the mechanisms of material removal, as well as surface integrity during nanogrinding. In one of their earliest studies [8], they conducted simulations with a single abrasive grain at two different orientations and discussed various important aspects of the nanometric abrasive simulation. Moreover in a later work [9], they simulated both ductile and brittle materials and studied not only the material removal mechanism and dislocation evolution, but also crack propagation for a silicon substrate. The effect of abrasive grain rake angle in nanometric grinding was studied by Komanduri et al. [10] with the aid of an appropriate MD model. More specifically in this work, the effect of rake angle on cutting forces and energy for a wide range of rake angle values from +45° to −75° was investigated with a rigid diamond tool. It was found that both cutting and thrust force increased for decreasing rake angle, with thrust force being mostly influenced as it increased almost 17 times, whereas a threefold increase was observed for cutting force; furthermore, the ratio of force components exceeded 1.0, with a value of 2.361 at a rake angle of −75°. Lin et al. [11] presented an MD nanogrinding model with a single hemispherical abrasive grain. Their study focused on material removal mechanisms and surface alterations of a silicon workpiece. With this model, they were able to observe chip formation and plastic deformation on the substrate and their analysis showed the correlation between grinding force fluctuations and dislocation movement.

In the relevant literature, there are also several computational studies with the use of MD method, related to high-speed grinding. Li et al. [12] investigated the effect of various process parameters such as grinding speed, depth of cut, and abrasive grain radius on chip formation and subsurface damage in high-speed grinding. Shimizu et al. [13] investigated the effect of grinding speed in relation to the wave propagation speed of the workpiece material with an MD model with a single hemispherical grain. It was found that, at lower speeds, increase of grinding speed resulted in lower plastic deformation but when the wave propagation speed was exceeded, severe hardening of the workpiece occurred. Li et al. [14] used an MD model to study the effect of grinding speed in nanoscale grinding of silicon and were able to determine the ductile to brittle cutting mode transition and the relation of stresses and subsurface damage. Guo et al. [15] presented an MD study of nanogrinding considering the effect of multiple passes of the abrasive grain on the workpiece. In the simulation, four passes of the abrasive grain were performed and after the determination of subsurface damage, it was concluded that the optimum depth of cut should not be higher than the half of the initial depth of cut.

Chen et al. [16] investigated the residual stresses during nanogrinding of silicon with a cylindrical tool. Simulations were carried out for various grinding depths from 5–30 Å. It was found that as the depth increased, although the region of transition between compressive and tensile residual stress remained almost the same, an increase of the compressive stresses near the surface was observed. Ren et al. [17] performed simulations regarding nanogrinding of monocrystalline nickel under various process conditions. Based on their findings, they observed an increase of cutting forces, potential, and temperature values with increased depth of cut and grinding speed values. Liu et al. [18] studied the effect of grinding speed and grinding depth on forces, stresses, temperature, and specific energy during nanogrinding of silicon carbide (SiC) with a conical tool. The increase of grinding speed and depth affected all quantities positively, with the greatest increase observed in the case of tangential grinding force and temperature. Liang et al. [19] presented a nanogrinding model with a cylindrical roller as cutting tool and investigated the influence of rotating speed and workpiece temperature on grinding forces. Higher rotational speed and increased initial workpiece temperature resulted in higher grinding forces.

Zhang et al. [20] investigated the surface damage layer formation during nanogrinding and its effect on workpiece mechanical properties. At first, they determined the optimum grinding speed for obtaining minimum thickness of damage layer and then performed simulations of tensile tests on the deformed workpieces. It was found that although Young’s modulus remained almost unaffected by the damage induced by nanogrinding, UTS was considerably affected, up to 47%. Xu et al. [21] also conducted a study regarding material removal and amorphous layer formation during nanometric grinding of a silicon wafer containing SiO_2_ region. They showed that, increase of grinding speed was able to reduce the damage layer depth and cutting forces, whereas friction coefficient increased with a large grinding depth. Ren et al. [22] performed nanogrinding simulations of monocrystalline nickel with a spherical abrasive grain and determined the effect of process parameters such as grinding speed, depth of cut, and abrasive grain radius on subsurface damage. At lower grinding speeds, stacking faults were formed in the substrate which were reduced in size gradually at higher grinding speeds and eventually vanished at 400 m/s. The increase of grinding depth and abrasive grain diameter led to an increase of stacking faults and dislocations, whereas the size of the subsurface damage layer was found to be almost unaffected by the grinding speed. The same scientific group, in a later study [23] investigated the effect of crystal orientation in the case of nanocrystalline nickel. After they carried out simulations for six different crystal orientations, they showed that burr height was minimum at (110)[$\bar{1}$10] direction, whereas the deformed layer depth was minimum at (111)[$\bar{1}$10] direction. Liu et al. [24] studied the effect of grinding speed and depth of cut on a SiC substrate. Their findings indicated that higher grinding speed leads to higher temperature but lower forces and lower thickness of amorphous layer, whereas higher depth of cut leads to higher temperature, higher forces, and higher thickness of amorphous layer.

Apart from silicon and common metals such as Cu, Al, and Ni, recently several studies focused also on the nanogrinding of bilayer workpieces. More specifically, Xu et al. [25] performed nanogrinding simulations for Cu-Si and Cu-SiO_2_ bilayer workpieces with a hemispherical tool. In their simulations, they employed various grinding depth and speed values and analyzed the deformation of the workpiece. Wang et al. [26] conducted a comprehensive study regarding nanogrinding of Al-Si bilayers under various grinding depth, speed, and abrasive grain radius values. They showed that an increase of the grain radius led to increase of workpiece temperature and grinding forces, and the same conclusions were drawn regarding grinding depth and speed. Moreover, the Si-Al bilayers exhibited higher forces and temperatures than the Al-Si bilayers. Fang et al. [27] investigated the material removal and surface integrity of Ni/Cu bilayers. Similarly to the study of Wang et al. [26], they found also that the increase of grinding speed, depth, and abrasive grain radius lead to increase of both grinding forces and temperature of the workpiece but it was also observed that the highest temperatures occurred in Ni/Cu bilayers, whereas the largest forces on simple Ni workpieces. Furthermore, lower abrasive grain radius and grinding depth led to better quality and lower damage. Finally, Xu et al. [28] also presented a thorough study on nanogrinding of Cu-Si substrates and examined the influence of copper layer thickness as well as grinding depth and speed on forces, temperature, potential and kinetic energy, and dislocation length, showing their correlations and their impact on phase transformation of the substrate as well.

In the previous studies, most models included a single abrasive grain at a linear motion. However, some researchers employed more sophisticated models regarding abrasive trajectory and shape. For example, Fang et al. [29] presented a model in which the abrasive grain moved at a complex trajectory, containing both a straight and an arc-shaped path. Eder et al. [30,31] developed a model for nanopolishing using cubic and spherical abrasive grains with random orientations while the workpiece surface was modeled using a pseudorandom Gaussian topography. Nevertheless, there is still a lack for models for peripheral nanogrinding using multiple abrasive grains.

Thus, in the present work, an MD model of peripheral nanogrinding is presented using multiple abrasive grains. The trajectory of the grains is consistent with the kinematics of peripheral nanogrinding and grains have different protrusion height as well. With the aid of this model, two sets of simulations are conducted; the first set of simulations is conducted in order to determine the effect of spacing between the two rows of abrasive grains on ground surface and chip formation, whereas the second set of simulations focuses on the effect of the rake angle of the abrasive grains on grinding forces, chip formation, and subsurface damage of the workpiece. In both cases, the results regarding the aforementioned quantities are discussed and conclusions on the effect of spacing on surface and chip formation as well as the effect of the negative rake angle on the outcome of the process are drawn.

## 2. Materials and Methods

**Figure 1:**
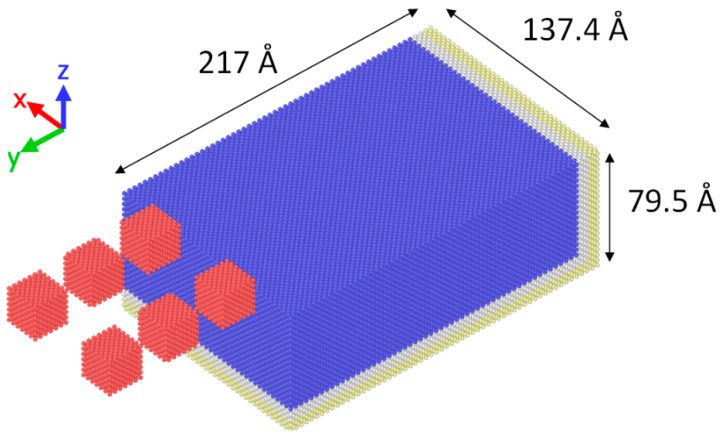
Schematic of the Molecular Dynamics (MD) model.

In the present study, an MD model regarding peripheral nanogrinding with multiple abrasive grains is presented. The aim of this study was the determination of the effect of spacing of the two rows of abrasive grains on surface and chip formation, as well as the effect of abrasive grain angle on grinding forces, friction coefficient, and subsurface damage. Thus, two different sets of simulations were carried out, with the necessary adjustments performed on the MD model. A general view of the model is depicted in Figure 1.

In the first set of simulations, 6 different spacing values were employed. For convenience, these values were defined in terms of the lattice parameter of tool material, denoted as a (a = 3.57 Å): 1a, 3a, 5a, 7a, 9a, and 11a. This wide range of values allowed for studying of considerably different cases in order to be able to determine more clearly the various states of the ground surface in respect to the process conditions. In each case, grinding length was 160 Å. The respective grains of each adjacent row had the same protrusion height and different position in the x axis, depending on the spacing in each case. The two rows of abrasive grains were situated in symmetrical positions in respect to the axis of symmetry of the workpiece in the x axis. The first set of abrasive grains, which were closest to the workpiece, was positioned initially at a depth of 2a and each following set was positioned 0.5a below the previous set of abrasive grains. In every case, the rake angle of the abrasive grains was 0°. For the second set of simulations, the same model was employed but the spacing was fixed at 3a, whereas the rake angle of the abrasive grains varied in the range of −5° to −60°. At each simulation, the rake angle of all abrasive grains was the same.

Workpiece material was monocrystalline copper, whereas the abrasive grains were diamond single crystal grains. The workpiece material was modeled with embedded atom model (EAM) potential function [32], which was particularly capable of describing the interactions due to metallic bonds as it takes into consideration the effects of electron density. For the interaction between abrasive grains and workpiece, Morse potential function for Cu-C pairs was employed [33]. The number of atoms for the MD model was 210,000. Furthermore, the dimensions of the copper workpiece along the three axes (xyz) was 217 Å × 137.4 Å × 79.5 Å.

As can be seen in Figure 1, the workpiece includes three different regions of atoms, namely, the region of Newtonian atoms, situated at the upper part of the workpiece and depicted in blue color, the region of thermostat atoms, situated below the Newtonian atoms region and depicted in white color, and the region of boundary atoms, situated at the right and bottom faces of the workpiece and depicted in beige color. The region of Newtonian atoms represents the main bulk of the workpiece, which is assumed to be deformable and atoms are allowed to move according to forces produced by their interaction with other atoms of the substrate or the atoms of the abrasive grains. The region of thermostat atoms is related to the regulation of workpiece temperature and dissipation of excessive heat by rescaling the velocity of these atoms [22] and finally, the region of fixed boundary atoms is essential to avoid rigid body motion and reduce the boundary effects. Moreover, periodic boundary conditions are imposed on the workpiece boundaries on the YZ planes. The diamond abrasive grains are considered much harder than the workpiece material and thus, are assumed to be a rigid body. For that reason, no interaction between C-C atoms is defined. The atoms of the workpiece are initially assigned velocities according to Maxwell-Boltzmann distribution based on the desired initial temperature and after a sufficient period for thermalization of the workpiece, nanogrinding takes place. The environment of the nanogrinding system is assumed to be vacuum, thus effects of grinding fluid or slurry are not taken into consideration.

The abrasive grains follow a complex path, including the influence of the rotation of the grinding wheel, which has a surface speed of 108 m/s and also incorporating the feed of the workpiece (v_f_), which is equal to 100 m/s, as the workpiece is fixed in the simulations. Feed direction is towards the –y axis, whereas rotation is taking place in the YZ plane. This complex movement is represented by a type of trochoid curve with the motion of the center or mass of each abrasive particle being described by Equations (1) and (2):

(1) $$x(t)=x_{0}+R\cos(\omega t+\varphi_{0})+v_{f}t$$

(2) $$y(t)=y_{0}+R\sin(\omega t+\varphi_{0})$$

In these equations, *x*_0_ and *y*_0_ represent the coordinates of the initial point where the center of mass of the abrasive grains was situated, R is the radius of the grinding wheel on which the abrasive grains are supposed to be placed, and ω is the radial velocity of the grinding wheel. The trajectory was determined in such a way that each abrasive grain loses contact with the workpiece only when it is close to the final grinding length. Initial temperature of the workpiece was set to 293 K and numerical time-step for the simulations was 1 fs. All simulations were carried out using LAMMPS software.

## 3. Results and Discussion

### 3.1. Effect of Abrasive Grains Spacing on Surface and Chip Formation and Subsurface Damage

**Figure 2:**
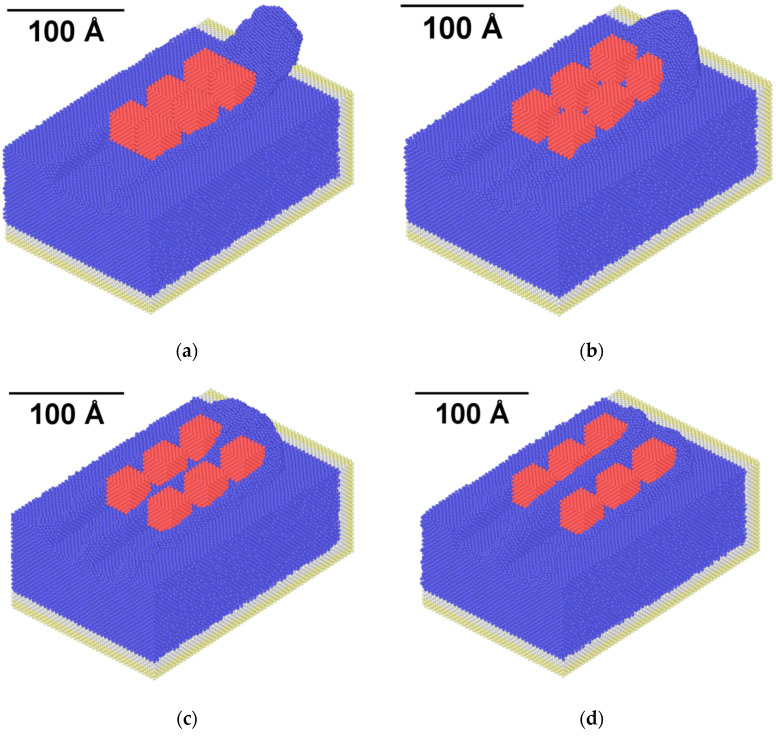

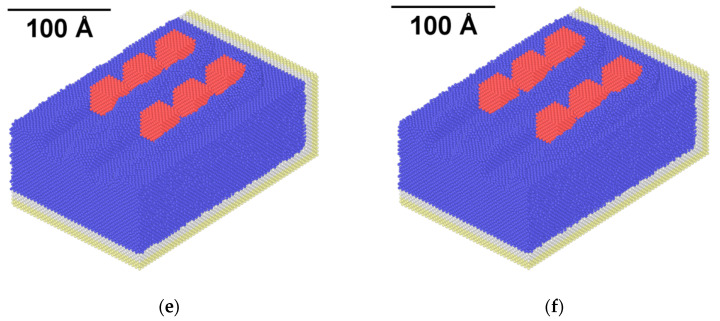
Snapshots of nanogrinding at the final timestep for the cases with spacing values of: (**a**) 1a, (**b**) 3a, (**c**) 5a, (**d**) 7a, (**e**) 9a, and (**f**) 11a.

**Figure 3:**
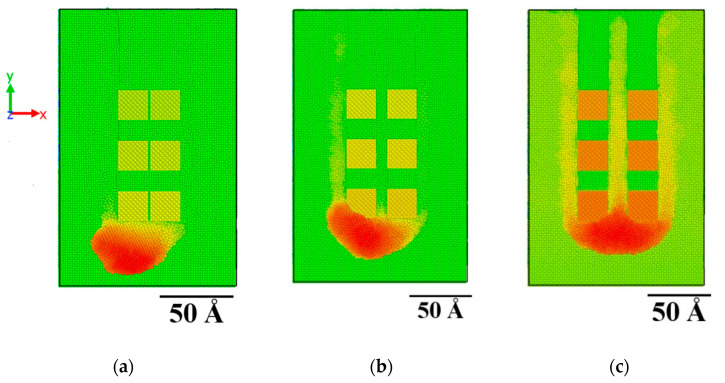

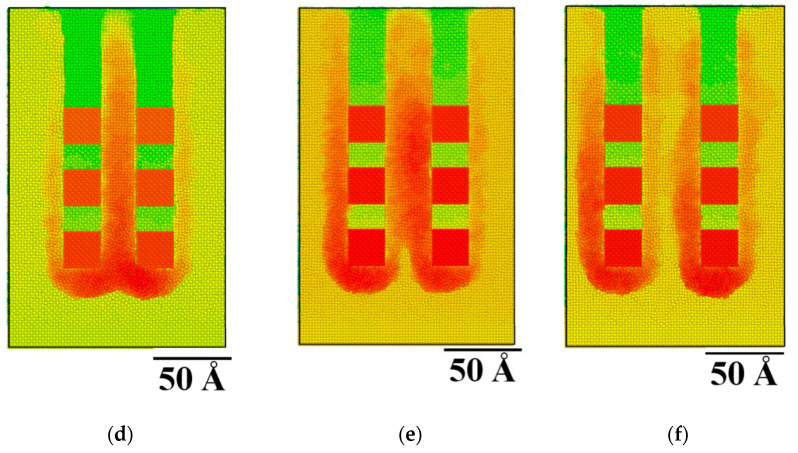
Top view of the workpiece at the final timestep for the cases with a spacing of: (**a**) 1a, (**b**) 3a, (**c**) 5a, (**d**) 7a, (**e**) 9a, and (**f**) 11a.

After the simulations were carried out, results regarding the first set of simulations will be discussed. The effect of spacing between the two rows of abrasive grains on the surface formed after the action of the abrasive grains and the produced chip can be observed in the snapshots of Figure 2. In this figure, snapshots of the last timestep of each case are being depicted for cases starting for spacing values from 1a to 11a. Moreover, in Figure 3, a top view of the workpiece at the same timesteps is presented with the atoms colored according to their z coordinate. From these results it can be considered that there are significant differences between these cases, something that will be examined in detail.

At first, it can be observed, from both Figure 2 and Figure 3, that due to the very close distance between the abrasive grains, a single groove is formed from the action of the two rows of abrasive grains, as their interaction with the workpiece material removes all atoms along their path, including atoms situated between them. As the spacing increases, gradually a zone of atoms is formed between the two rows, and for spacing values from 5a and beyond, two separate grooves exist. However, the chip produced by the material removal from both rows of abrasive grains is still common until spacing value exceeds 7a. In these cases, the chip produced by each row of abrasive grains is separate. Apart from the chip in the front of the abrasive grains, there is also an amount of atoms deposited on the area between the two grooves and on the sides of the grooves, especially in the cases with larger spacing.

**Figure 4:**
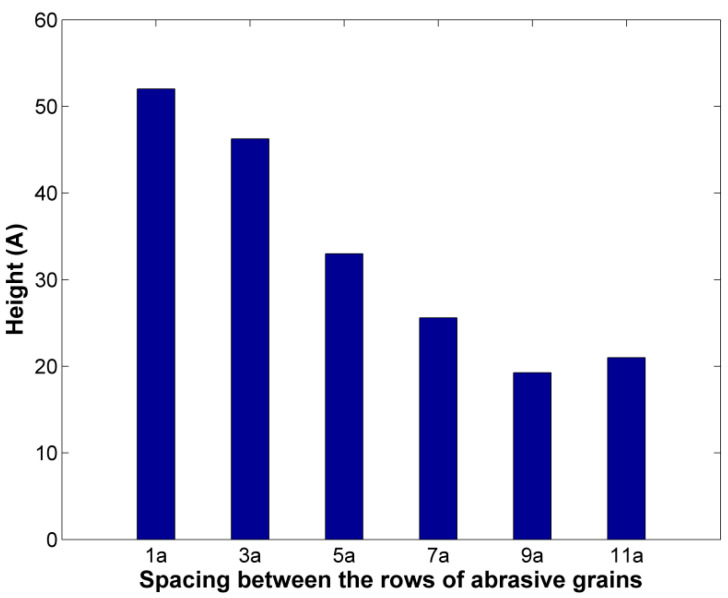
Height of chip in cases with different spacing between the rows of abrasive grains.

In order to quantify the effect of spacing on chip dimension, the chip height was also measured in each case, as can be seen in Figure 4. It is to be noted that the chip height is calculated from the top of the initial workpiece surface. The chip height is shown to become constantly lower as spacing increases before eventually stabilizing around 100 Å. From these observations, it can be concluded that the largest reduction of chip height actually coincides with the beginning of the formation of two separate grooves and then, stabilization of maximum chip height occurs after the chips produced by the two adjacent rows of abrasive grains are separated.

**Figure 5:**
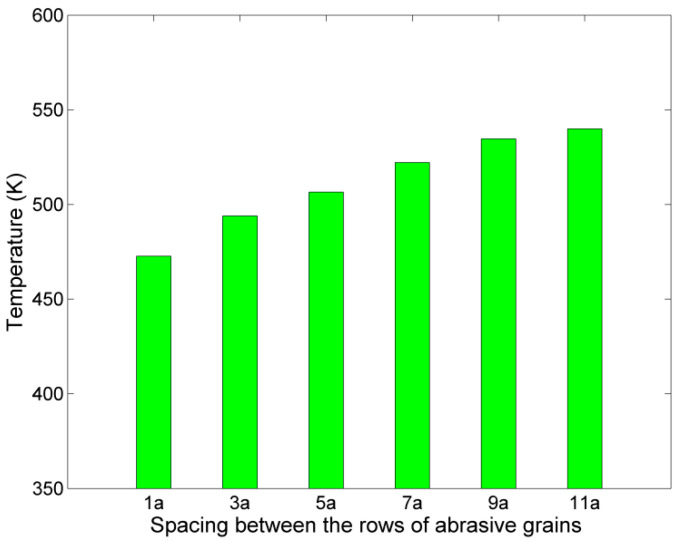
Average temperature of Newtonian atoms in cases with different spacing between the rows of abrasive grains.

The observations from the snapshots of nanogrinding and chip height measurements can be further supported by the computed values of average temperature in the region of Newtonian atoms. As the spacing becomes larger, it can be seen from Figure 5 that average temperature values are gradually rising from a temperature of 472.65 K for a spacing of 1a and eventually, they stabilize at 530–540 K. Temperature is directly connected with variations in the kinetic energy of the atoms and thus, an increase of temperature indicates either that the atoms which are affected by the grinding action have increased velocities or that more atoms are affected by the action of the abrasive grains. As the process parameters are the same but the larger spacing between the grains leads to a wider affected area, the second case is more probable. What is more interesting, the most abrupt changes in the average temperatures coincide with the cases which mark the transitions from the formation of a single groove to the formation of separate grooves and finally, separate pileups of atoms, i.e., after the cases with a spacing of 3a and 7a, respectively.

The results of this set of simulations can be useful for the determination of optimum grinding strategy towards the rendering of a high-quality flat surface on the workpiece. For example, when the spacing between the adjacent rows of abrasive grains is larger, a large amount of material remains between the rows of grains and different process conditions and number of passes in x axis are required to render a flat surface, than in a case where almost no material is left between the grains. On the other hand, narrower spacing between the grains leads to material removal in a smaller area. Thus, in fact in order to determine the strategy required to achieve the highest efficiency, a detailed study based on the findings of the present work should be conducted.

### 3.2. Effect of Abrasive Grain Rake Angle on Grinding Forces, Chip Formation, and Subsurface Damage

After the results regarding the first set of simulations were presented and discussed, the analysis of the results of the second set of simulations is taking place. In this set of simulations, the effect of abrasive grain rake angle is investigated for negative values starting from −5° to −60°. Although no similar works regarding the rake angle of abrasive grains are reported, this range of values was selected in accordance with the relevant literature on nanocutting simulations with negative rake angle tools [10,34,35,36,37,38,39,40,41,42,43,44,45,46,47,48]. More specifically, most authors have chosen negative rake angle values up to −45° [34,43,45] or −60° [10,35,38], and Lai et al. [37] pointed out that the critical rake angle for formation of chip is −65°, as they proved that higher negative rake angles resulted in plastic deformation of the substrate without a pileup of atoms.

**Figure 6:**
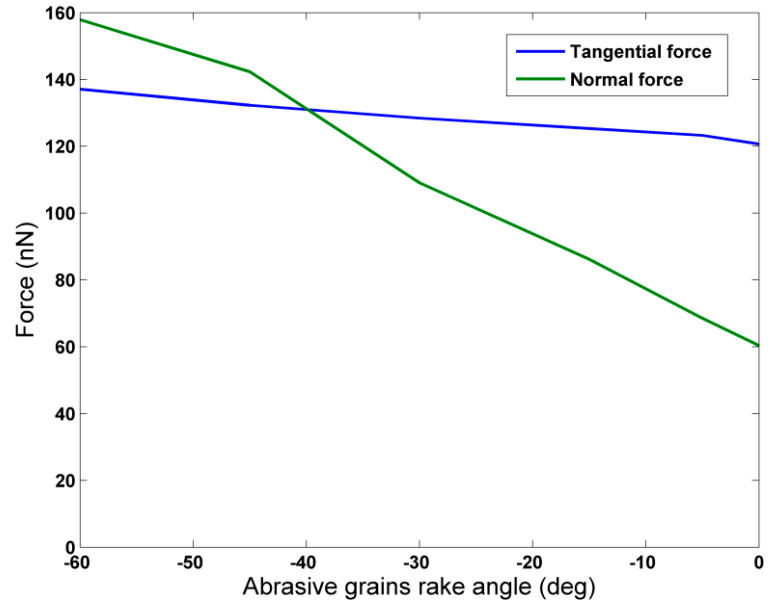
Grinding force components in respect to abrasive grains rake angle.

In Figure 6, the tangential (F_y_) and normal (F_z_) grinding force components values are depicted, in respect to different rake angle values. It should be noted that F_x_ values are not presented as there is no motion along this axis and force component values are negligible, compared to the two other force components. From Figure 6, it can be seen that both F_y_ and F_z_ values increase as rake angle values increase in magnitude and eventually, F_z_ values become greater than F_y_ values. The increasing trend for both force components is consistent with every relevant work in the literature [10,34,35,36,37,38,39,40,41,42,43,44,45,46,47,48]. Moreover, a slower increase is attested for F_y_ than for F_z_, as F_z_ values increase 2.62 times, whereas F_y_ values increase only 1.14 times, as it was also observed in several works, such as [38,39,43,48].

**Figure 7:**
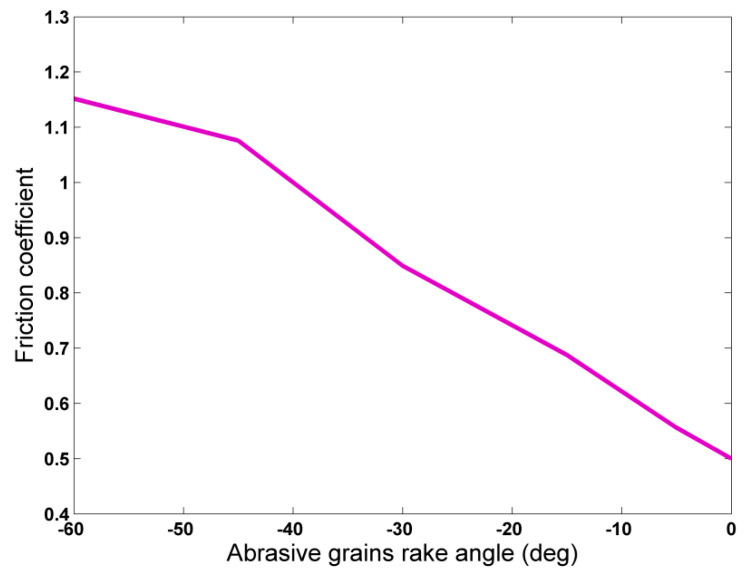
Average friction coefficient values in respect to abrasive grains rake angle.

In Figure 7, the average friction coefficient values are depicted in respect to the rake angle of the abrasive grains. As in most relevant studies, the friction coefficient is calculated as the quotient of normal and tangential grinding force. From Figure 7, it can be seen that friction coefficient values increase as the magnitude of the rake angle increases. Friction coefficient value exceeds 1.0 above almost −40° and eventually reaches the value 1.15 for rake angle value of −60°. In the relevant literature, several authors have also reported friction coefficient values over 1.0 in the case of negative rake angle values in nanocutting such as [38,39,43] and nanogrinding [10], with maximum values being 1.6, 1.4, 1.55, and 2.361, respectively. However, regarding the rake angle value above which the normal force exceeds the value of the tangential one, the researchers’ findings do not always agree. Among the authors who reported values of normal force greater than the values of tangential force, Komanduri et al. [38] determined the critical rake angle value at almost −30° similar to Tong et al. [39], whereas Pei et al. [43] and Alhafez and Urbassek [46] calculated a value near −45° and Komanduri et al. [10] calculated a value of −15° in simulations of nanogrinding. Moreover, although Promyoo et al. [41,47] studied only positive values of rake angle over 0°, the maximum friction coefficient was found at 0° in both cases with a value of almost 0.4 and 0.886, respectively, with an increasing trend of friction coefficient as rake angle was decreased. Thus, the value predicted in the current work is in compliance with these works. On the other hand, Dai et al. [34,40] reported higher values of tangential force at least until −45° and Zhao et al. [36] reported a value of 0.55 at −40°. The reason for these variations is perhaps that friction coefficient is dependent on other process parameters to some extent, such as cutting speed, depth of cut, tool radius, and so, simulations conducted under different values of these parameters yield different results on the critical rake angle value which leads to μ > 1.0.

**Figure 8:**
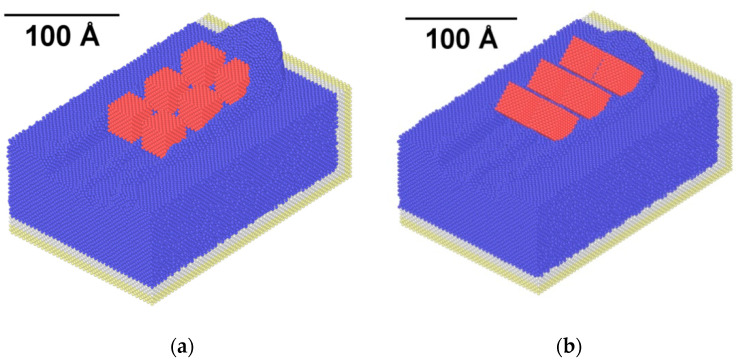
Snapshots of nanogrinding with abrasive grain rake angle of: (**a**) 0° and (**b**) −45°.

**Figure 9:**
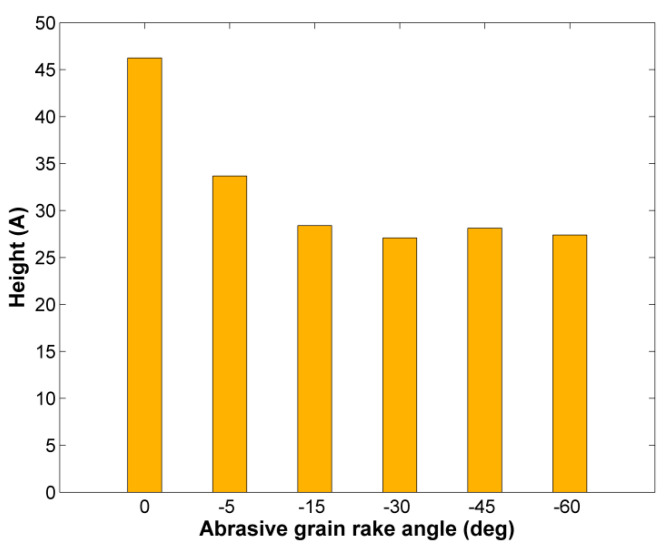
Chip height during nanogrinding in respect to abrasive grain rake angle.

Regarding the dimensions of the chip produced during nanogrinding, it is expected that different abrasive grain rake angle will produce significant alterations to the chip. In Figure 8, the chip morphology in the final snapshots of two representative cases of the second set of simulations, namely, at a rake angle value of 0° and −45° are compared, and in Figure 9, the height of the pile of the removed atoms is depicted in each case. It can be directly seen from Figure 9 that the height of the chip is considerably reduced for rake angle values up to −30° and then it remains practically stable. The reduction of chip height, with higher negative rake angle was also observed in several works in the relevant literature [34,35,36,37,38,40,43] due to the increased compression of the removed atoms by the abrasive grain face which contacts them at a highly inclined configuration.

In order to investigate the rake angle effect on subsurface damage, as well, analysis was performed by computing centrosymmetry parameter (CSP) values. According to CSP value for each atom of the Newtonian region of atoms, these atoms can be classified into five categories: for CSP < 3, the atoms belong to the original Face-centered Cubic (FCC) crystal; for 3 < CSP < 5, the atoms belong to a partial dislocation; for 5 < CSP < 8, the atoms belong to a stacking fault; and higher values of CSP indicate surface and surface edge atoms [49].

**Figure 10:**
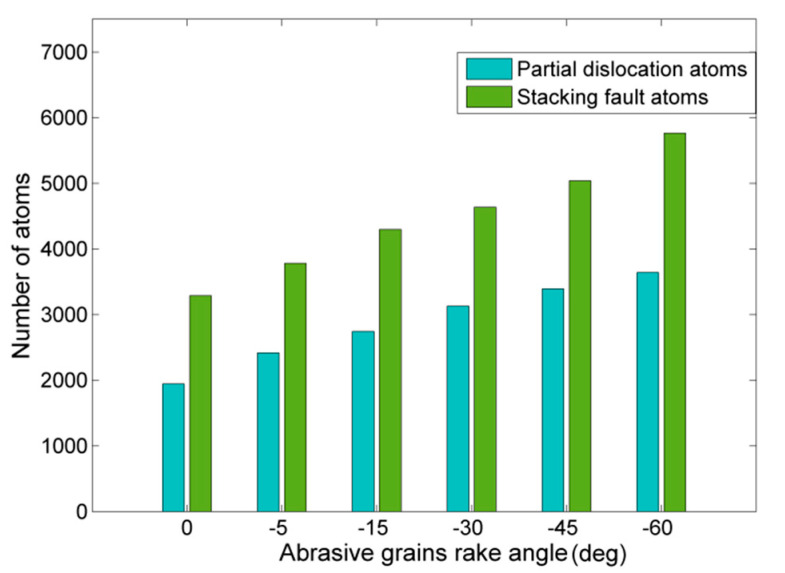
Number of atoms pertinent to partial dislocations and stacking faults in respect to abrasive grains rake angle.

The results depicted in Figure 10 indicate that an increase of abrasive grains rake angle leads gradually to more atoms of the workpiece being part of stacking faults or partial dislocations. Thus, grinding with highly negative rake angle grains leads to more intense plastic deformation of the workpiece as the imposed pressure on it becomes larger and material removal becomes gradually more difficult. A few works in the relevant literature also indicate this trend in nanocutting processes. Dai et al. [34,40] observed variations in the number of atoms pertinent to intrinsic stacking faults [34] during nanocutting of copper or in the number of atoms with different coordination during nanocutting of silicon [40]. Lai et al. [37] found out that plastic deformation was more intense in the workpiece with a decrease of rake angle towards highly negative values and that a lot of dislocations were concentrated in the subsurface area for these rake angle values. Similarly, Zhang et al. [45] computed higher dislocation densities for negative rake angles than for positive ones and Alhafez and Urbassek [46] identified a clear change of mechanism of plasticity at high negative rake angles, which was also related to the suppression of chip formation by thoroughly studying the dislocation movement.

## 4. Conclusions

In the present work, an MD model for nanogrinding with multiple abrasive grains was developed in order to investigate the effect of spacing between the adjacent rows of abrasive grains on ground surface and chip formation as well as the effect of abrasive grains rake angle on grinding forces, chip formation, and subsurface alterations. From the analysis of the results, several conclusions were drawn.

Regarding the effect of spacing of abrasive grains, it was shown that it affects considerably the formation of the ground surface and chip. Narrower spacing leads to the formation of a single groove in each pass and chip height is large, as chip is mainly concentrated in front of the abrasive grains. As spacing increases, due to more material remaining in the area between the rows of the abrasive grains, two separate grooves are formed with a common pileup and finally, there is even no interaction between the atoms removed from each groove, as separate pileups are formed. Chip height and average temperature measurements exhibit trends compatible with these observations and thus, support the qualitative findings.

Regarding the effect of abrasive grain rake angle, according to the findings, a direct correlation between grinding forces and rake angle was determined, with both tangential and normal grinding force increasing with higher negative rake angle values. The normal component of grinding force was more affected by the rake angle as it increased almost threefold when rake angle was −60° in comparison to 0°. At the same time, the friction coefficient increased to values over 1.0 for rake angles higher than −40° as higher friction forces occur to the more intense contact of abrasive grains and workpiece surface. Finally, analysis of centrosymmetry parameter values indicated an increase of plastic deformation in the workpiece for higher negative rake angle values, probably due to the higher pressure exerted on the workpiece surface. These results are important as they are consistent with trends observed also in the micro- and macroscale grinding and can provide an insight into phenomena which either cannot be observed in the experiments or cannot be captured with continuum method such as FEM.
